# Visual Search of Experts in Medical Image Reading: The Effect of Training, Target Prevalence, and Expert Knowledge

**DOI:** 10.3389/fpsyg.2013.00166

**Published:** 2013-04-05

**Authors:** Ryoichi Nakashima, Kazufumi Kobayashi, Eriko Maeda, Takeharu Yoshikawa, Kazuhiko Yokosawa

**Affiliations:** ^1^Department of Psychology, The University of TokyoTokyo, Japan; ^2^The University of Tokyo HospitalTokyo, Japan

**Keywords:** radiologists, visual search, lesion search task, skill development, prevalence effect, multiple-target search, expert knowledge

## Abstract

The aims of this study are (a) To determine the effect of training on the multiple-target lesion search performance; and (b) To examine the effect of target prevalence on the performance of radiologists and novices. We conducted four sessions of 500 trials in a lesion search on a medical image task in which participants searched for three different target lesions. Participants were 10 radiologists and novices. In each session, the prevalence of the different target lesions varied from low (2%) to high (40%). The sensitivity of novices was higher in the later sessions than in the first session, whereas there were no differences among sessions in radiologists. The improvement on sensitivity of novices was largely due to attenuations of false alarm (FA) errors. In addition, miss rates of the three targets did not differ in data of novices, whereas radiologists produced a higher miss rate for the highest prevalence target lesion (non-serious lesion) than for the other two lesions (serious lesions). The conclusions are (a) The training for the multiple-target lesion search task can be effective to reduce FA errors; and (b) The prevalence effect on lesion search can be attenuated by the multiple-target identification and the knowledge about seriousness of lesions. This suggests that acquired knowledge about normal cases and serious lesions is an important aspect of a radiologists’ skill in searching for medical lesions and their high performance levels.

## Introduction

People termed “experts” show remarkably higher performance than novices, especially in their domain of expertise (Bédard and Chi, 1992, for a review). For example, there are professional proofreaders (Asano et al., 2008), chess masters (Chase and Simon, 1973), aviation security screeners (Schwaninger et al., 2005), and so on. Among them, we focused on radiologists (i.e., experts for medical image reading). Previous studies reported that radiologists and cytologists appear to be better than novices at recognizing images from their specialized field (Evans et al., 2011a), and radiologists have good sensitivity to targets in x-ray images (Sowden et al., 2000).

What makes people experts in a domain? One factor is that experts usually conduct a task in a particular domain under the situation where there are reward for detecting targets, and this leads to a high (and optimal) performance (e.g., Maddox, 2002; Navalpakkam et al., 2009, 2010; Hickey et al., 2010). For example, for radiologists who are the expert of medical screening, discovery of a serious lesion should be rewarding, because a life is saved (e.g., Brawley and Kramer, 2005), thus they can detect lesions very well.

Another factor involves training (and experience). In addition to acquiring knowledge about a particular domain, experts have typically undertaken many trials, on a daily basis, in which they performed a given task in this domain (e.g., Nodine et al., 1996, also see Chase and Simon, 1973). Training and experience can influence on the performance, because experienced radiologists generally show a higher performance than interns or resident in medical image reading task (e.g., Parasuraman, 1986; Nodine et al., 1999, 2002).

The present study examined the effect of training in a particular skill on the performance. Specifically, it is important for medical students (initially novices) to develop the ability to accurately detect lesions (i.e., become expert in radiological search tasks), because expertise in this domain is highly correlated with the ability to save lives of patients. In previous studies (e.g., Parasuraman, 1986; Nodine et al., 1999, 2002), although participants differed with respect to stages in their careers, all participants could be classified as experts in their domain, not novices. Therefore, it is not clear how novices come to acquire expertise in a particular domain through training.

In this study, we focused on the skill pertaining to a medical lesion search task in medical image. The medical image reading (e.g., medical screening) generally involves two basic processes: detection of targets and interpretation (Krupinski, 2010). The detection processing where radiologists find some candidates of lesion could be very similar to visual search task where observers find a target among distractors (e.g., Treisman and Gelade, 1980).

Typical visual search tasks in experimental psychology require observers to search for a single type of initially specified target, i.e., at onsets of an experimental trial or session (e.g., Treisman and Gelade, 1980). Some previous studies have suggested that training improves visual search performance in a complex scene, however, in most of these tasks, participants were required to search only for a single type of target (e.g., Sowden et al., 2000; McCarley et al., 2004). For example, Sowden et al. (2000) reported novices’ detection sensitivity improved with training in a task requiring detection of a low-contrast dot detection task. In addition, McCarley et al. (2004) reported that training improved accuracy and speed of target detection in a visual search task involving the image of a knife depicted in baggage from an x-ray screening protocol.

A normal medical lesion search task, contrastingly, differs significantly from search tasks used in these preceding studies. It requires that radiologists simultaneously search for multiple-target lesions rather for one particular lesion (e.g., Barbaum et al., 2010). Generally these target lesions are not visually similar to each other. Typically, tasks that require searches for several different kinds of targets are relatively difficult, as indexed by additional cognitive costs that lower overall performance relative to single-target searches (e.g., Menneer et al., 2004, 2007, 2009). Yet, at least in some multiple-target search tasks, which involve simple target stimuli (i.e., alphanumeric characters), there is evidence that multiple-target costs can be attenuated by practice (e.g., Kaplan and Carvellas, 1965). However, the characteristics of visual search tasks differ between tasks using simple stimuli versus those employing naturalistic stimuli. That is, visual searches of natural scenes may afford more efficient searches than visual searches using simple stimuli, due to the presence of scene-specific forms that guide attention to certain regions of search array (Wolfe et al., 2011). Recent study discussed that a medical image serves as an equivalent to a natural scene image (Drew et al., 2013). Accordingly, it remains unclear whether or not training actually improves performance when participants engage in more realistic tasks (i.e., tasks using naturalistic stimuli) that require search for one of several (visually different) targets.

A further practical problem that surrounds medical screening (i.e., lesion search task) is that target prevalence varies depending on the type of target. Generally, very serious lesions that can develop into a fatal illness (e.g., cancer) do not appear very often in daily medical screening (Benard et al., 2004). Some previous studies (e.g., Wolfe et al., 2005, 2007) have shown that observers are surprisingly poor at finding rare targets in visual searches, termed the *prevalence effect*. The prevalence effect also raises a most important issue for medical screening because it implies that visual search experts will often miss targets, especially rare (and serious) target. Clearly this is a grave implication. Adding to this concern, Evans et al. (2011b) recently reported that radiologists (i.e., experts of visual search for lesions in medical image) may indeed miss rate target lesions more often than frequent target lesions in a lesion search task.

In summary, two aims of this study are as follows. First, we determined the effect of training on the multiple-target search performance in a medical image reading task (i.e., visual search for lesions in medical images). Second, we examined the effect of target prevalence in the multiple-target search task by comparing the performance of experts (radiologists) with that of novices.

## Materials and Methods

We conducted a visual search task in which participants simultaneously searched for three types of target lesions. Four experimental sessions of 500 trials were conducted to examine the effect of training. Participants were novices and radiologists, allowing for an examination of acquired skill differences in the performance of these two groups. Our primary interest was to investigate how performance of novices approaches to that of visual search experts (radiologists) during training, thus the results of experts may be served as baseline (i.e., maximum) performances that performances of novices should get to in that sense. Our secondary interest was to compare the performance between novices and experts for the three types of target lesions. We manipulated the prevalence of the different target lesions from low (2%) to high (40%), to examine this issue.

In this experiment, we used a lesion search task in which computed tomography (CT) images contained one of three types of target lesions: Bulla, ground-glass nodule (GGN), and cancer. The seriousness of each lesion is different from the others. If a single bulla exists in the lung, it is not medically meaningful. Radiologists often do not mention it, even when they find it. GGN can sometimes represent early stage cancer, and typically radiologists will follow up such a lesion closely when they find it. Cancer is a very serious lesion and can develop into a fatal illness. Radiologists always closely examine the lesion and then request that physicians provide treatment.

The lesions could be visually distinguished by color: Bulla as a black circle, GGN as a gray circle, and cancer as a white circle. Furthermore, all lesions were discriminatively larger than the background blood vessels, thus it is possible for even novices to distinguish the target lesions from distracters (i.e., blood vessels). This manipulation made the experimental task relatively easy, because this task might be similar to the feature search task (e.g., Treisman and Gelade, 1980). We used this easy task, because our primary aim is to observe the performances of novices and we want novices who were unfamiliar with medical images to recognize the target lesions correctly.

Overall, targets occurred on 50% of trials: Bulla prevalence was 40%, GGN was 8%, and cancer was 2% of trials. The order of target frequencies was similar to those of the lesions in real medical searches. This experimental condition is very similar to that in Experiment 3 (“mixed condition”) of Wolfe’s et al. (2007) study, in which the prevalence structure was target A on 34% of trials, B on 10%, C on 5%, D on 1%, and no target was presented on the remaining 50% of trials. In Wolfe’s study, although a statistical analysis was not conducted, the results nonetheless showed rare targets were missed more often than frequent targets.

On any given trial, there was at most one target lesion. Therefore, in this experiment, we need not consider a “satisfaction of search” phenomenon (e.g., Samuel et al., 1995; Fleck et al., 2010), in which observers often miss the second target once they detect one target even when two targets are presented simultaneously.

### Participants

Ten radiologists from the University of Tokyo Hospital (age: 26–41 years; 2–16 years of experience in the interpretation of chest CT), and 10 novice young adults (age: 21–23 years) completed this experiment. Although one might wonder that 10 participants seem to relatively few, there are many previous studies using about 10 participants to examine visual search performance (e.g., Treisman and Gelade, 1980; Wolfe et al., 2011), effect of training (e.g., Sowden et al., 2000; McCarley et al., 2004), and medical image reading performance (e.g., Nodine et al., 1999, 2002). All had normal or corrected-to-normal vision. The experiment was approved by the institutional review board (IRB of Graduate School of Medicine, The University of Tokyo) and written informed consent was obtained from all participants.

### Stimuli and apparatus

**Figure 1:**
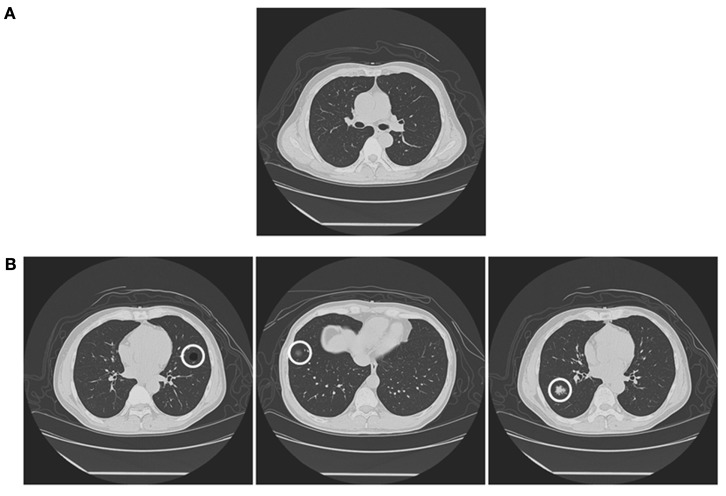
**Samples of stimuli in the experiment**. **(A)** A background CT image (target-absent image), and **(B)** target-present images, including a bulla-present image (Left), a GGN-present image (Center), and a cancer-present image (Right). Each target lesion is marked by a white ring in this figure. Note; the white ring was not presented in the experiment.

We prepared 250 CT images of lungs (15° × 15° of visual angle). These were image slices of healthy lungs (Figure 1A), which included no lesions. We used each image eight times (2000 background CT images).

Three types of target lesion images were prepared (Figure 1B): Bulla, GGN, and cancer (about 0.8° × 0.8°). As described above, bulla looked like a black circle, GGN a gray circle, and cancer a white circle. We prepared various images of each lesion. To create a target-present image, we inserted one lesion image onto one of the background CT images. We added bulla on 800, GGN on 160, and cancer on 40 CT background images. We allocated target positions carefully to prevent spatial biases. The target lesions were located at the plausible locations where all lesions can exist. The remaining 1000 background images were target-absent images. We divided the 2000 images into 4 equivalent groups, each of which included 200 bulla-presented images, 40 GGN-presented images, 10 cancer-presented images, and 250 lesion-absent images. Radiologists in the University of Tokyo Hospital, who did not participate in the experiments, supervised stimulus construction.

Presentation of stimuli and response recording were controlled by Matlab software, using the Psychophysics Toolbox extensions (Brainard, 1997; Pelli, 1997). Stimuli were displayed on a 22″ monitor (1024 × 768 pixels). Participants viewed the monitor from a distance of 77 cm (fixed by a chinrest) in a dark room.

### Procedure

Participants completed four sessions of 500 trials each. Each session included 250 target-present (200 bulla-present, 40 GGN-present, and 10 cancer-present trials) and 250 target-absent trials. One stimuli-group was assigned to one session randomly for each participant. On each trial, a fixation (500 ms) and then blank display (500 ms) were presented, followed by a search display (i.e., a CT image). The search display was presented until participants responded or after 1000 ms. Although a display was presented for, at most, 1000 ms, onset of the next trial was contingent on participant’s response. The time limit for a search display presentation was used to encourage quick responses.

Novices and radiologists were initially provided with information covering the visual properties of the targets. It should be noted that all radiologists could recognized the names of lesion targets when presented their visual images, and we told them the names of target lesions. Participants were instructed to search for a target (any one of the three types) and to respond by pressing the appropriate (labeled) key to identify the target that was presented or all targets were absent as accurately as and then as quickly as possible. They were informed that there was at most one target lesion in a given trial. No participants knew the prevalence structure of target lesions. Trial order in each session was determined randomly.

## Results

Outliers in the Reaction Time (RT), defined as RTs less than 200 ms or more than 4000 ms, were removed from the analysis (0.15% of trials in radiologists and 0.53% in novices). Although, participants were instructed to identify the target types, our primary interest was not to examine the performance of target discrimination in this study. Thus, for analysis in this study, we used a stringent definition of miss errors: Miss Trials were the trials in which participants responded “target-absent” within each of three target lesion present trials.

First, we calculated a signal detection measure of sensitivity *d*′ in both groups (Table 1). Overall, performances in both novices and radiologists were relatively high. A two-way mixed factorial ANOVA with Group as a between-participants factor and Session as a within-participants factor was conducted. The interaction between Group and Session was significant, *F*(3, 54) = 3.45, *p* < 0.03, $\eta_{p}^{2}$ = 0.16. The sensitivity of novices was higher in the later sessions than in the first session, *p*s < 0.001, whereas there were no differences among sessions in radiologists, *p*s > 0.4. This indicates that the effect of training on visual search performance can work instantly in this task. Although the sensitivity of novices increased, it did not reach the level exhibited by expert radiologists even in the final session, *p* < 0.05. Thus, the sensitivity in radiologists was consistently high and reliably greater than levels exhibited by novices, *F*(1, 18) = 11.73, *p* < 0.003, $\eta_{p}^{2}$ = 0.39. The main effect of session was significant, *F*(3, 54) = 6.70, *p* < 0.001, $\eta_{p}^{2}$ = 0.27, reflecting that improvement occurred in novices.

**Table 1:** **The summary of results in the experiment of (a) novices and (b) radiologists**.

	Session 1	Session 2	Session 3	Session 4
**(A)**				
*d*′	3.17 (0.22)	3.67 (0.30)	3.80 (0.23)	4.06 (0.31)
Miss rate (%)	5.32 (1.05)	5.85 (1.42)	6.52 (1.98)	5.35 (1.39)
FA rate (%)	8.96 (2.75)	4.10 (1.30)	2.04 (0.44)	1.96 (0.57)
RT (ms), target-present trials	877 (65)	780 (41)	742 (43)	703 (26)
RT (ms), target-absent trials	1199 (107)	1005 (96)	916 (70)	848 (56)
**(B)**				
*d*′	4.51 (0.17)	4.65 (0.11)	4.47 (0.14)	4.72 (0.16)
Miss rate (%)	3.49 (0.79)	3.37 (0.84)	3.89 (0.65)	3.21 (0.73)
FA rate (%)	0.60 (0.20)	0.24 (0.09)	0.44 (0.16)	0.24 (0.09)
RT (ms), target-present trials	807 (25)	730 (19)	721 (26)	723 (31)
RT (ms), target-absent trials	1011 (56)	840 (47)	819 (45)	780 (47)

*Standard errors are in parentheses*.

Table 1 also shows miss rates and false alarm (FA) rates. Mean miss rates in novices were higher than in radiologists, although the difference was not significant, *F* < 1.6. More importantly, session did not affect miss rates in either group, *F* < 1.1. In contrast, FA rates attenuated as sessions progressed from the first to the last session, *F*(3, 54) = 11.36, *p* < 0.001, $\eta_{p}^{2}$ = 0.39, and FA rates in novices were higher than in radiologists, *F*(1, 18) = 24.24, *p* < 0.001, $\eta_{p}^{2}$ = 0.57. Further, the interaction was significant, *F*(3, 54) = 7.35, *p* < 0.001, $\eta_{p}^{2}$ = 0.29, indicating that the FA rates of novices were lower in later sessions than in the first session, *p*s < 0.001, whereas there were no differences among sessions with radiologists, *p*s > 0.6.

In this experiment, each target-absent image was presented in each session. To examine the possibility that repeating target-absent image presentation would cause the attenuation of FA errors, we divided the data in first session of novices into two groups, in which no target-absent images were presented repeatedly: data in first half and second half. Miss rates were not different (first half: 7.0% vs. second half: 7.5%), *t*(9) = 0.52, *p* > 0.6. FA rate was lower in second half (5.7%) than in first half (12.2%), *t*(9) = 3.27, *p* < 0.01.

To accomplish the second aim of this study, namely the examination of the target prevalence effect in the performance of novices versus experts (radiologists), we divided the miss rate data of novices and radiologists into three groups based on the target types (bulla: 40%, GGN: 8%, and cancer: 2% target prevalences) and compared them. Results appear in Table 2. These data were collapsed over the four sessions, because, as described above, there were no differences among miss rates as function of session. To compare the outcomes, we first arc-sine transformed the miss rate data {*y*′ = arcsin[sqrt(*y*)]} to compensate for unequal variances in the data (Hogg and Craig, 1995), because the number of trials for these three targets differed. We conducted a two-way mixed factorial ANOVA with Group as a between-participants factor and Target type as a within-participants factor. The interaction between Group and Target type was significant, *F*(2, 36) = 6.10, *p* < 0.01, $\eta_{p}^{2}$ = 0.25. Miss rates of the three targets did not differ in data of novices, *p*s > 0.4, whereas, the miss rate did vary as a function of target type for radiologists. Specifically, radiologists produced a higher miss rate for bulla lesions (i.e., the highest prevalence target lesion) than for the other two lesions, *p*s < 0.04. Although the mean miss rate of bulla was slightly higher in novices than in radiologists, this difference did not reach significance, *p* > 0.4. Miss rates of GGN and cancer, both of which were low prevalence target lesions, were lower in radiologists than in novices, *p*s < 0.03. The performance for these two lesions led to the result that overall miss rate was lower in radiologists than in novices, *F*(1, 18) = 6.53, *p* < 0.02, $\eta_{p}^{2}$ = 0.27. The main effect of Target Type was not significant, *F*(2, 36) = 1.68, *p* > 0.1.

**Table 2:** **The summary of miss rates of target lesions collapsed over the sessions**.

	Bulla (%)	GGN (%)	Cancer (%)
**(A)**			
Novices	6.68 (1.38)	8.68 (2.61)	11.51 (5.34)
Radiologists	4.31 (0.79)	1.70 (0.43)	1.50 (0.67)

*Standard errors are in parentheses*.

Overall RTs (Table 1) were shorter on target-present trials than on target-absent trials, *F*(1, 18) = 27.11, *p* < 0.001, $\eta_{p}^{2}$ = 0.60. Moreover, RTs became shorter as sessions progressed in both groups, *F*(3, 54) = 36.37, *p* < 0.001, $\eta_{p}^{2}$ = 0.67. The difference between two groups was not significant, *F*(1, 18) = 1.51, *p* > 0.2.

## Discussion

### The effect of training on medical image reading task

The overall finding that the RTs were longer on target-absent trials than on target-present trials is a typical result in visual search tasks (Chun and Wolfe, 1996). In both groups, the speed of responding improved as sessions progressed, showing a general practice effect on visual search (cf. Chun and Jiang, 1998).

Mean *d*′ of radiologists and novices were relatively high (above four in radiologists and above three in novices in Session 1). High *d*′ indicates that, in addition to the ease of task, participants detected the target as correctly as possible following the instruction faithfully. Detection sensitivity of radiologists was not influenced by the training. This is likely due to a ceiling effect because the average values of *d*′ for radiologists was very high. The finding that sensitivity of radiologists was higher than novices confirms a general expectation that radiologists would show very high performance in their domain of expertise (e.g., Sowden et al., 2000; Evans et al., 2011a). In contrast, *d*′ values for novices increased from Session 1 to 4. This is consistent with the results of previous studies (Sowden et al., 2000; McCarley et al., 2004). Therefore, the training in which participants receive many trials can generally be effective to improve their performance. That performance of novices failed to match that of radiologists even in the final session implies that while practice helps visual search, the amount of training received in 2000 trials is insufficient; much more trials may be required for novices to perform as well as radiologists.

We also discovered that the profiles of miss and FA error rates over sessions (cf. Table 1). This differs from reports of FA profiles in previous studies (Sowden et al., 2000; McCarley et al., 2004). For example, McCarley et al. reported that, in a single-target search task simulating an aviation security task, the improvement of performance with training derived mainly from error attenuations reflected in miss rates, not FA rates. In contrast, this study found that training actually attenuated FA rate, not miss rate in the medical lesion search task. Menneer et al. (2009) has also reported that FA rates were reduced more than miss rates as a result of training (although this was not discussed in detail). Thus, the training may generally reduce FA errors. However, considering that the reduction of FA errors means that observers come to recognize the target-absent images correctly, this result points toward a possible explanation with special importance for medical lesion search task: The training (i.e., receiving many trials of a task) can be effective for gaining knowledge about normal cases of medical images (i.e., target-absent images).

One reason of this could be that the task was a multiple-target search task. In a single-target search task (e.g., McCarley et al., 2004), observers only have to retain one target representation and find the visual object matches this representation. Therefore, learning of the visual properties of the target is effective in improving the visual search performance. In contrast, in a multiple-target search task, observers must retain multiple representations of targets, and this is an additional cognitive cost for visual search (Menneer et al., 2009). In this case, it is relatively difficult to learn the visual properties of all the targets. Especially in medical lesion search tasks, it may be easier to obtain knowledge of a normal search displays (e.g., CT images of healthy lungs) and detect, as a target, anything that violates the “normality” of a CT image as established by the trials which contained no lesions. To obtain knowledge of normal cases, it is necessary to compare normal cases (target-absent images) and abnormal cases (target-present images). Thus, we noted that only viewing many target-absent images would be insufficient for acquiring this knowledge.

Another reason for differences in the findings of previous studies and those reported in this study may derive from task differences. McCarley et al. (2004) used an aviation security task whereas the present study used a medical lesion search task. Generally, in an aviation security tasks the objects to-be-identified are arranged randomly within a suitcase whereas in a lesion search on medical image task the arrangement of objects is more confined. For instance, the structure of a lung (or other organs) can be well-defined, thereby limiting the possible locations of objects within this organ. Accordingly, in the former type of task, the recognition of a global search display may not be useful in achieving successful target detection. By contrast, in medical lesion search tasks, the structure of a particular organ, such as a healthy lung, can be effective to detect target lesions, because global information of image can guide an attentional allocation by implying the possible location of lesions. Recent studies reported that visual search in a natural scene, which usually has a well-structured layout of information, is facilitated after observers pre-viewed the scene (e.g., Hollingworth, 2009; Võ and Henderson, 2010; Castelhano and Heaven, 2011). Novice participants may gain the knowledge about normal search displays (i.e., CT images of healthy lungs), because the contextual information of the global image is useful to detect target lesions.

In sum, because the task is a multiple-target search task, and there is a well-defined structure of a lung (or other organs), training for the medical lesion search can be effective to obtain knowledge of normal search displays (e.g., CT images of healthy lungs). This is consistent with the speculation that expert radiologists bring greater knowledge of what can be considered “normal” in a chest x-ray image (Myles-Worsley et al., 1988).

In many cases, experts show very high performances especially in their domain of expertise, not so high in the other domains (e.g., Chase and Simon, 1973; Sowden et al., 2000; Evans et al., 2011a; but see Asano et al., 2008). Thus, for the effective skill learning in a specific domain, it is important that people are given a training by the tasks in the domain.

Comparing the results of this study with those of McCarley et al. (2004), we speculate that miss or FA errors can be attenuated separately by the training in different situations. Miss errors may be attenuated when it is important to obtain the knowledge about target (e.g., a single-target search, visual search display containing randomly distributed objects). In contrast, FA errors may be attenuated when it is essential to obtain the knowledge about the whole display (e.g., a multiple-target search, well-structured visual search display). It is necessary to examine the generalization of this speculation in the future researches.

In some search tasks in our daily lives such as medical screening tasks or aviation security tasks, it is not generally acceptable to miss targets because these errors have serious consequences. Thus, it is very important to reduce miss errors in the tasks. However, simply producing more “target-present” responses is not a practical solution to minimize miss errors. In this case, FA errors will automatically increase. In medical screening, higher FA errors require much more time and labor of radiologists, because they should examine “target-present” images carefully to characterize the lesion and determine its diagnosis, even if “target-present” is actually false. That is, there are problems that derive from heightened FA errors in daily visual searches. In this study, we showed that the training is effective in reducing FA errors, in spite of the fact that observers (i.e., novices) did not understand the importance of attenuating FA errors. This implies that training can be an effective means of instilling knowledge about normal cases, although it may have an effect on the other factors such as becoming familiar with visual images of lesion. Such knowledge is an essential element of expertise, especially in medical lesion search task.

### The effect of target prevalence in medical screening task

In our daily life, there are some visual searches for rare targets especially in the professional screening tasks such as medical screenings (i.e., search for lesions) or airport security screenings (i.e., search for dangerous tools). As described in the Introduction, very serious lesions (e.g., cancer) are relatively rare occurrences in daily medical screenings. Some previous psychology studies (e.g., Wolfe et al., 2005, 2007) have reported that rare targets are missed more often than frequent targets in a visual search task. Thus, the issue of prevalence effect is critical for medical lesion search because in a medical context this effect would pose serious societal problems and lead to a major negative impact on the medical patients.

To examine the prevalence effect in medical screening tasks, we divided the miss rate data into three sets one set for each target lesion. In contrast to previous studies, in this study we did not obtain results indicating that low target prevalence increases the miss errors, even in novices. Although this experiment was similar to “Experiment 3” in Wolfe et al. (2007), which showed that rare targets are missed more often than frequent targets, our results do not agree with that finding. What accounts for the difference in results between these two studies? One reason may be that the present task appeared to be relatively easy in that overall errors were relatively low. However, we suggest here that the primary cause of these divergent results resides in procedures which required different methods of responding on the part of participants. In Wolfe’s experiment, participants were told to judge whether a target was present or absent (i.e., a target detection task). In the present study, on the other hand, participants were instructed to identify either a detected target or to report that all targets were absent. In other words, the present task required target identification. One of the main grounds of prevalence effect is a strong response bias favoring the “target-absent” response in the low prevalence condition (Fleck and Mitroff, 2007; but see Van Wert et al., 2009); in fact, low prevalence errors have been shown to vanish in a target identification task where this bias was prevented (Rich et al., 2008). This implies that a multiple-target search-and-identification task can be effective in attenuating the prevalence effect. Practically speaking, radiologists generally search for multiple types of lesions simultaneously during routine medical screenings. Thus, the task that experts perform on a daily basis can itself reduce the miss errors of lesions.

Based on the results of novices, the suggestion that rare targets are not missed more often than frequent targets in a target identification task (Rich et al., 2008) can be applied to medical screening tasks, where observers view a realistic image rather than simple alphabetical stimuli. In addition, the results of radiologists establish another noteworthy finding. In contrast to the prevalence effect, radiologists actually missed rare targets *less often* than other types of targets. This cannot be explained simply in terms of the influence of a particular identification task. It is more likely due to the fact that the frequency of target types co-varied with the seriousness of a diagnosis. In this regard this experiment validly simulates real medical screening in which lower target prevalence indicates more serious lesions. Specifically, bulla is not a meaningful lesion, whereas GGN and cancer are medically serious lesions. Therefore, the knowledge of the importance and seriousness of lesions can be effective in motivating the avoidance of errors (misses) during lesion searches, even though the lesions are rare targets. Presumably, radiologists acquire this knowledge of the targets from extensive experience, and not simply as a result of instructions about the importance of targets (e.g., names of targets). That is, it had been shown that novice observers often show relatively high miss rates of rare targets even when they have received instructions that it is very important to detect the rare targets (Rich et al., 2008).

A comparison between the performance of novices and radiologists supports the preceding suggestion. The miss rate of bulla, which was present in 40% of trials, was not different between two groups. Radiologists showed better performance only when they detected serious lesions. Based on this result, we suggest that not only the multiple-target identification task but also the knowledge about the seriousness of lesions can be important to improve the performance in medical image reading. Experts have more visual knowledge in their expert domain than novices in a medical screening (Evans et al., 2011a), or an airport security screening (Schwaninger et al., 2005). Thus, to obtain expert knowledge can generally be effective to improve the visual search performance, especially the visual search for rare targets, in the expert domain.

### The limitation and implications for future research

Novices must become experts through training on a daily basis in which they acquire knowledge of their domain. The knowledge of normal (lesion free) cases is not enough to turn novices into experts, because this does not explain the fact that miss errors did not attenuate with training in this experiment. Further, the results that experts showed higher performance when they detect serious targets indicate that the knowledge of seriousness of targets, which is not obtained by the visual search training, is also important to improve the performance in medical screening task. The effect of factors other than training should be examined in detail in future research.

We suggest that training of observers in a multiple-target search task which presents a well-structured visual display (e.g., an organ structure) can be effective in obtaining knowledge of normal search displays. However, the generality of this claim remains to be verified because the degree to which it applies to other types of search tasks or even other medical image reading tasks is unclear. Perhaps the superior skills displayed by these particular experts in this relatively easy task are specific to the present study. Furthermore, in medical image reading, there are two basic processes: detection and interpretation (Krupinski, 2010). We examined only the detection process but, of course, the interpretation process is also important. These issues are crucial to fully understand the effect of training on skill learning, and it is essential to examine these issues further in future research (Perhaps, it may be necessary to conduct large research studies).

## Conclusion

In this study, we examined the effect of extensive training on the medical lesion search performance. The experimental task in this study simulated the normal medical lesion search tasks in which radiologists search simultaneously for multiple-target lesions in a well-structured image display. Therefore, daily searches such as medical screenings is effective in obtaining knowledge about normal cases (i.e., target-absent images). It is an important element of expertise which can explain attenuation of FA errors, because the ability to recognize a normal CT image is essential for radiologists who sometimes detect abnormalities in a single glance by fixating (or detecting) a lesion that does not “fit” into a normal configuration (Kundel and Nodine, 1975). In addition, multiple-target identification task can be effective to attenuate the prevalence effect in visual search (Rich et al., 2008). Further, the knowledge of importance of targets (e.g., seriousness of lesions) can be effective to reduce miss errors of rare target lesions. Some of the suggestions (e.g., multiple-target search task, or knowledge about the importance of targets), at least, can be applied not only to medical lesion search task but also the other visual search tasks.

## Conflict of Interest Statement

The authors declare that the research was conducted in the absence of any commercial or financial relationships that could be construed as a potential conflict of interest.
